# Human corneal stromal stem cells support limbal epithelial cells cultured on RAFT tissue equivalents

**DOI:** 10.1038/srep16186

**Published:** 2015-11-04

**Authors:** Alvena K Kureshi, Marc Dziasko, James L Funderburgh, Julie T Daniels

**Affiliations:** 1 Ocular Biology & Therapeutics, Institute of Ophthalmology, University College London, London, United Kingdom; 2 Department of Ophthalmology, UPMC Eye Centre, University of Pittsburgh, Pittsburgh, Pennsylvania, USA

## Abstract

Human limbal epithelial cells (HLE) and corneal stromal stem cells (CSSC) reside in close proximity *in vivo* in the corneal limbal stem cell niche. However, HLE are typically cultured *in vitro* without supporting niche cells. Here, we re-create the cell-cell juxtaposition of the native environment *in vitro*, to provide a tool for investigation of epithelial-stromal cell interactions and to optimize HLE culture conditions for potential therapeutic application. RAFT (Real Architecture For 3D Tissue) tissue equivalents (TEs) were used as a 3-dimensional substrate for co-culturing HLE and CSSC. Our results demonstrate that a monolayer of HLE that maintained expression of p63α, ABCB5, CK8 and CK15 (HLE markers), formed on the surface of RAFT TEs within 13 days of culture. CSSC remained in close proximity to HLE and maintained expression of mesenchymal stem cell markers. This simple technique has a short preparation time of only 15 days with the onset of HLE layering and differentiation observed. Furthermore, co-cultivation of HLE with another niche cell type (CSSC) directly on RAFT TEs, eliminates the requirement for animal-derived feeder cells. RAFT TEs may be useful for future therapeutic delivery of multiple cell types to restore the limbal niche following ocular surface injury or disease.

The epithelial stem cells (LESC) of the cornea reside in the limbus; a microenvironment or ‘niche’ that plays a crucial role in regulating the fate of these cells^1^. LESC divide, differentiate and migrate centripetally from the limbus towards the central cornea^2^. This is essential for maintaining a healthy and transparent cornea enabling normal vision^3^. The LESC population in the adult cornea may become deficient with any ocular insult caused by injury, infection or immunological diseases such as Stephens-Johnsons syndrome or pemphigoid. Patients with LESC deficiency suffer from loss of a healthy epithelium causing corneal vascularization, inflammation and conjunctivalisation of the cornea^4^ impairing visual acuity and causing partial or complete blindness.

A variety of factors within the limbal niche are thought to influence/control the fate of LESC activity. These include physical interactions of cells with their extracellular matrix (ECM) such as ECM geometry and mechanical properties. The surrounding cells including stromal cells, vascular endothelial cells and melanocytes in addition to soluble factors in the ECM, are thought to facilitate molecular cross-talk which act as cues for the maintenance or differentiation of LESC phenotype. Recently, a population of mesenchymal stem cells (MSCs) has been identified in the superficial limbal stroma of the human cornea^5^. These corneal stromal stem cells (CSSC) are located in close proximity to human limbal epithelial cells (HLE) within the limbal niche^6^ and it is thought that they may support the potency of epithelial stem cells *in vivo*^5^,^7^. Our recent studies have shown that CSSC can now be isolated reliably from the limbal stromal niche of organ-culture corneas, facilitating the culture and *in vitro* expansion of CSSC to large quantities that would be useful for bioengineering an epithelial-stromal tissue equivalent or in development of a cell-based therapy for treatment of ocular surface defects^8^.

To restore function of the epithelium in patients with LESC deficiency, it is important to replenish the LESC population and possibly also CSSC as supporting niche cells. Current stem cell therapies include the use of amniotic membrane or alternative substrates to culture HLE *ex vivo* prior to transplantation^9^,^10^,^11^. Importantly, these approaches do not include CSSC. We have previously presented the use of RAFT tissue equivalents for the culture and expansion of HLE for possible transplantation^12^. Up until now, RAFT TEs were seeded with limbal fibroblasts and it was shown that the presence of stromal cells supported the growth and multi-layering of HLE. However, these previous methods have all been dependent on the use of murine 3T3 fibroblasts for *in vitro* expansion of HLE. This traditional approach typically lengthens the culture time and poses a risk of transfer of adventitious agents to the patient. Ideally, an LESC therapy would eliminate the use of murine 3T3 fibroblasts and be quick to manufacture, cutting down the time taken from bench to bedside. Multiple grafts for testing safety and efficacy should also be prepared to satisfy requirements imposed by the regulating authorities^13^.

In this study, we proposed that RAFT TEs could be used as a 3-dimensional (3D) substrate to cultivate a mixed population of HLE with CSSC, without the use of animal-derived feeder cells. Importantly, it could also serve as a biomimetic 3D model of the limbal niche to investigate epithelial-stromal cell interactions.

## Results

### Morphology of CSSC and HLE in 2D

**Figure 1:**
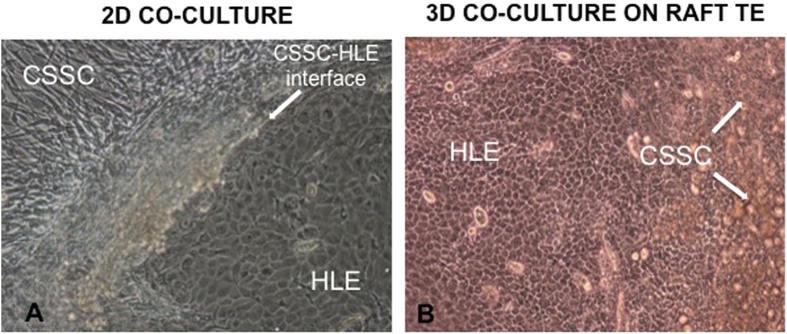
(**a**) Light microscopy image showing a primary culture of a mixed population of human corneal stromal stem cells (CSSC) and human limbal epithelial cells (HLE) on plastic (passage 0, day 15). Spontaneous organization of the two different cell types is observed (CSSC-HLE interface). The characteristic cobblestone morphology of a HLE cluster is visible adjacent to confluent CSSC at its periphery (magnification ×10). (**b**) Light microscopy image showing a mixed population of human corneal stromal stem cells (CSSC) and human limbal epithelial cells (HLE) cultured on RAFT TE for 13 days. A confluent monolayer of epithelial cells with the characteristic cobblestone morphology is visible with clumps of CSSC (white arrows) shedding from the surface of RAFT TE (magnification ×10).

CSSC and HLE cell populations thrived in close proximity to each other in 2D co-culture. CSSC became confluent quickly and changed morphology from their small square appearance to longer spindle-shaped cells. At the early stages of culture, small HLE colonies surrounded by CSSC appeared in culture. These smaller colonies had the characteristic cobblestone appearance of HLE cells and merged into one larger colony with CSSC only remaining at the periphery by the later stage of culture (Fig. 1a).

### Morphology of CSSC and HLE in 3D—on RAFT TEs

**Figure 2:**
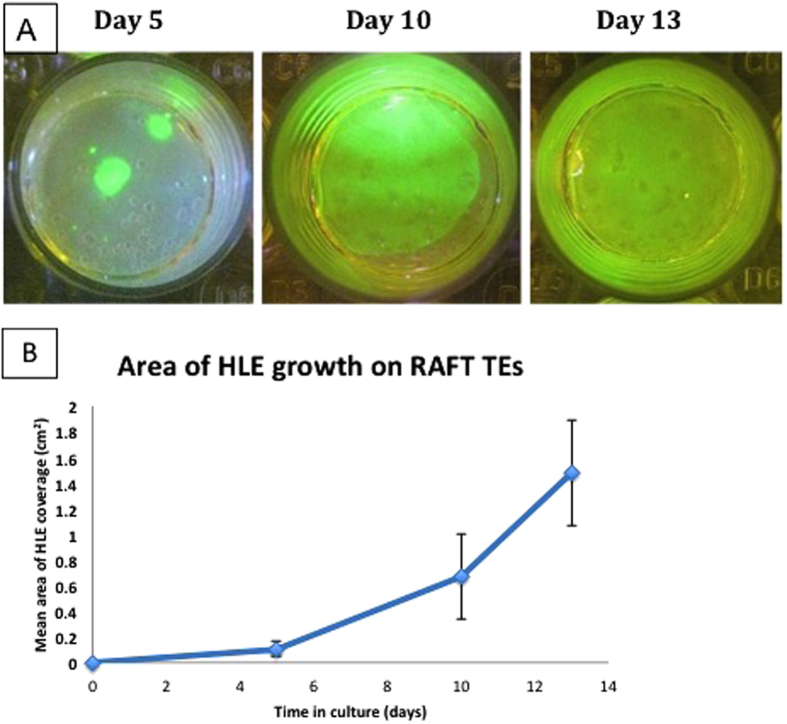
(**a**) Photographs of fluorescein diacetate (FdA)-stained RAFT TEs with mixed population of CSSC and HLE on surface. Images are taken with a camera using a yellow lens under a blue light. HLE growth (seen in green) increases over time in culture until a confluent monolayer is achieved by day 13. (**b**) Graph illustrating mean area of HLE growth (n = 4 donors) over 13 days of culture. RAFT TEs were stained with FdA and images taken at different time points during culture. Each point represents a mean value taken from the average of 4 biological donors (i.e. 4 RAFT TEs per donor).

Multiple small colonies of HLE appeared after 2 days of culture. These expanded quickly and merged into a larger HLE colony with the characteristic cobblestone appearance (Fig. 1b) that covered the surface of RAFT TEs as a monolayer of cells within 13 days of culture (Fig. 2a). The majority of CSSC appeared to be pushed towards the edges of RAFT TEs by the expanding colony of HLE, eventually shedding off in to the media (Fig. 1b).

### Measurement of HLE growth on surface of RAFT TEs (FdA staining)

Photographs of fluorescein diacetate-stained RAFT TEs were taken at day 5, 10 and 13 in culture (Fig. 2a) and analysed using Image J software to assess the extent of HLE growth over the culture period. 3D co-cultures on RAFT TEs demonstrated an increase in mean area of HLE growth from 0.11 ± 0.06 cm^2^ at day 5 to 0.67 ± 0.33 cm^2^ by day 10 and 1.48 ± 0.41 cm^2^ by day 13 (mean ± SD) (Fig. 2b).

### Expression of HLE markers on RAFT TEs

**Figure 3:**
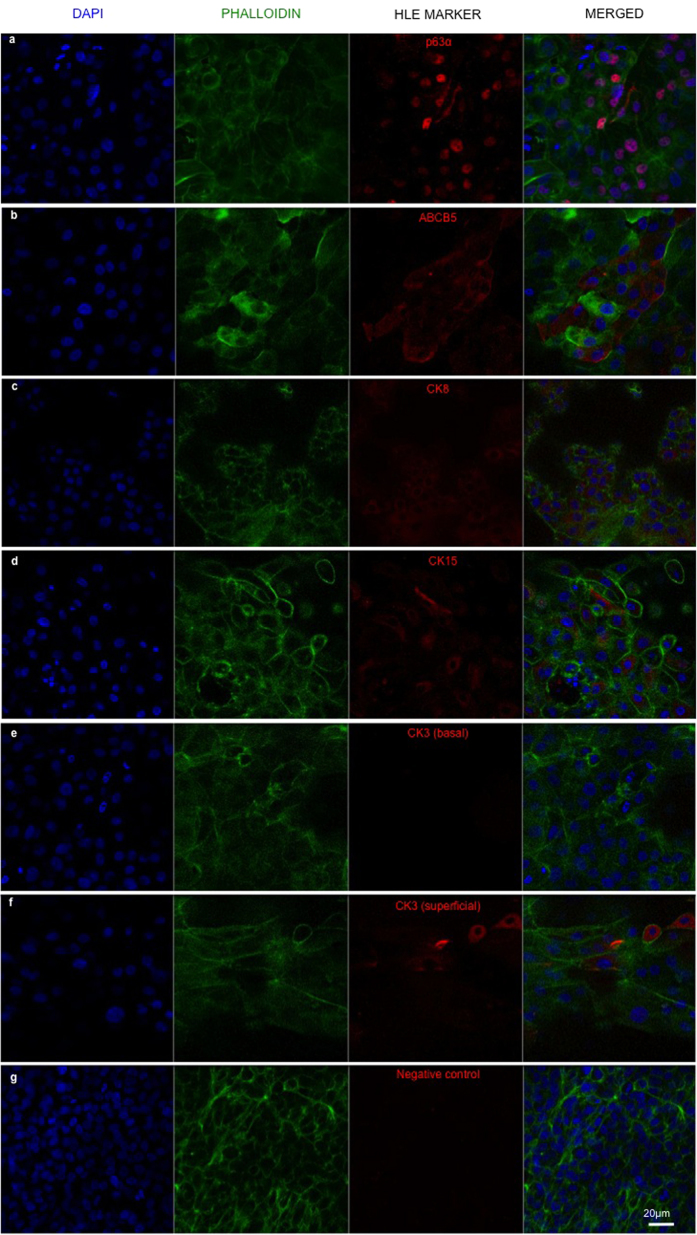
Confocal split channel images of RAFT TEs cultured with a mixed population of CSSC and HLE for 13 days in CSSC media. Positive staining of HLE markers (**a**) p63α, (**b**) ABCB5, (**c**) CK8 and (**d**) CK15 can be seen in red; (**e**) basal HLE do not express HLE differentiation marker, CK3; (**f**) CK3 expression sparsely observed in superficial HLE; (**g**) negative control without primary antibody. (Blue—DAPI; green—phalloidin). Scale bar 20 μm.

RAFT TEs at day 13 of culture were assessed using immunohistochemistry for expression of a range of HLE markers. Positive expression of p63α and ABCB5 (putative epithelial stem cell markers), CK8 and CK15 (Fig. 3a–d respectively) was observed in a large proportion of basal HLE, confirming the HLE phenotype and indicating some cells retained their progenitor potential throughout culture. As expected, HLE differentiation marker, CK3, was not expressed in basal HLE (Fig. 3d) but a few larger, superficial HLE exhibited positive expression of CK3, indicating the onset of HLE differentiation. A negative control where the primary antibody was excluded demonstrated very low levels of fluorescence (Fig. 3g).

### Expression of CSSC markers on RAFT TEs

**Figure 4:**
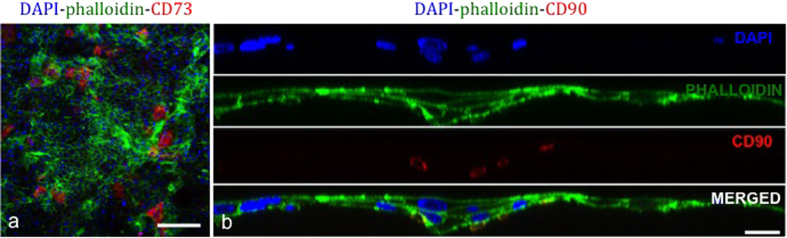
Confocal images of RAFT TEs cultured with a mixed population of CSSC and HLE for 13 days in CSSC media. Positive staining of CSSC (mesenchymal markers) (**a**) CD73 expression of CSSC sparsely remaining on surface of RAFT TE in close proximity to HLE (scale bar 200 μm); (**b**) Split channel line scan showing cross-section of RAFT TE at day 13 of culture. CSSC with positive CD90 expression (seen in red) appear burrowed beneath basal HLE. (Blue - DAPI; Green - phalloidin). Scale bar 20 μm.

RAFT TEs were assessed with immunohistochemistry for mesenchymal stem cell (MSC) markers CD73 and CD90. Due to their highly proliferative nature, the majority of CSSC shed off in to the media as they became too confluent. However, it appeared that a few CSSC remained on the surface and expressed MSC markers CD73 (Fig. 4a) and CD90, (Fig. 4b) confirming the CSSC phenotype.

### Interaction of CSSC and HLE on RAFT TEs—Confocal microscopy analysis

Confocal microscopy analysis of RAFT TEs revealed that a small population of CSSC remained on the surface of RAFT TEs, supporting the growth of HLE. CSSC had smaller, irregular shaped nuclei when compared to HLE and thus could be identified from their morphology (Fig. 4b). A small number of CSSC that expressed CD90 appeared to burrow beneath the basal layer of HLE (Fig. 4b).

### Interaction of CSSC and HLE on RAFT TEs—TEM analysis

**Figure 5:**
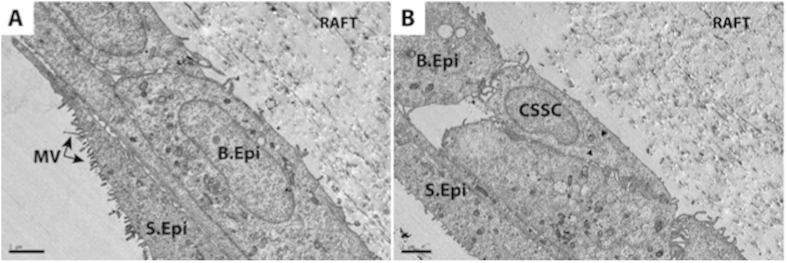
TEM micrographs of RAFT TEs cultured with a mixed population of CSSC and HLE for 13 days. (**a**) multilayering of HLE with a basal epithelial cell (B.Epi) visible beneath a superficial epithelial (S.Epi) cell with typical microvilli (MV) features on the apical surface. (**b**) reveals the close interaction of a CSSC and a basal epithelial cell (B.Epi) on the basal layer. CSSC can be distinguished from HLE as it appears smaller than HLE with a more spindle morphology. Black arrows indicate presence of endoplasmic reticulum, which is more developed in CSSC and thus more visible. Scale bar 2 μm.

TEM analysis of RAFT TEs demonstrated CSSC were in close contact with HLE (Fig. 5). Electron micrographs of a cross-section of RAFT TEs cultured with a mixed population of cells at Day 13 illustrate the proximity of CSSC with basal epithelial cells (B.Epi). Superficial epithelial cells (S.Epi) appear stratified with microvilli visible on the apical surface. CSSC are distinguished from HLE by a more apparent presence of endoplasmic reticulum and spindle morphology.

## Discussion

This study has demonstrated a reproducible, 3D co-culture method to support the cultivation of HLE using CSSC on RAFT TEs. These biomimetic substrates could be used as a 3D *in vitro* model of the limbal niche with which to explore mesenchymal-epithelial cell interactions within an anatomical context.

Collagenase digestion of human corneal limbus was used to isolate a mixed population of HLE with CSSC (mesenchymal stromal stem cells). Chen and colleagues^14^ also utilized a collagenase-digestion method and demonstrated that limbal digestion with collagenase, but not dispase, could isolate both limbal epithelial progenitors and closely associated mesenchymal stromal cells. The close association of these two cell types was shown to be crucial for promoting epithelial clonal growth^15^ but also important for maintaining the stem cell phenotype of both cell populations *in vivo*^6^,^16^,^17^. In our co-cultures, expression of ABCB5; a limbal epithelial stem cell marker required for corneal repair and development^18^, together with other HLE markers; CK8^19^ and CK15^20^, indicated the HLE phenotype had been maintained. Similarly, CSSC phenotype was also maintained and confirmed by expression of mesenchymal stem cell markers CD73 and CD90. We have previously demonstrated successful isolation of CSSC from the limbal niche of long-term stored organ culture corneas^8^. These are more readily available than fresh Optisol-stored corneas and provide a useful population of mesenchymal stromal stem cells for development of cell therapies.

The contribution of mesenchymal stromal cells in maintenance of the limbal epithelial stem cell phenotype in 2D culture has previously been demonstrated^21^,^22^. Studies by Ainscough *et al.*^23^ found that stromal cells isolated from the limbus were superior in their function to support HLE *in vitro* than cells from the cornea or sclera. Other studies have presented 3D culture methods using a culture insert membrane to culture HLE. Nakatsu and colleagues^24^ isolated human limbal mesenchymal stromal cells (LMCs) from the deep stroma to compare their efficiency at expanding HLE with 3T3 feeder cells. These were cultured on the opposite side of a culture insert membrane to single HLE or HLE clusters, which prevented contamination by feeder cells. They found that LMCs were equally efficient at supporting HLE expansion than 3T3 feeder cells in 3D culture. A similar study by Li and colleagues^25^ demonstrated that limbal niche cells from the superficial stroma were more efficient at maintaining limbal epithelial cells than limbal stromal cells obtained from the deep stroma. In addition, a recent study by our lab demonstrated that 2D cultures of HLE cells in CSSC media, with or without 3T3 fibroblasts, were more successful and more organized than those cultured in the traditional method^22^. These studies highlight that the close proximity of stromal/niche cells to HLE is beneficial for the *in vitro* expansion of HLE. However, as HLE were cultured on a culture insert membrane or on plastic, these would have to be dissociated once confluent, if transferred to a patient for transplantation.

By using RAFT TEs, we were able to successfully recreate this juxtaposition of two cell types seen in the native limbal niche. This can be a useful 3D model for investigating epithelial-stromal cell interactions but also enables creation of an ocular surface tissue equivalent for the possible transplantation of a cell therapy for LESCD treatment. Cultured LESC therapies are routinely transplanted in isolation of any supporting niche/feeder cells that may effect efficacy and hinder their long-term survival in the host. Hence, it may then prove beneficial to transplant supporting niche cells such as CSSC, together with epithelial cells for improved function and long-term survival in the host.

Our 3D co-culture method using RAFT TEs has many advantages and overcomes many challenges faced by existing culture methods. This improved technique is faster than previous methods that have used RAFT TEs incorporated with limbal fibroblasts as a culture substrate for HLE cultivation^12^,^26^. Limbal fibroblasts are typically grown by explant culture, which can take several weeks to grow before the required cell density is achieved. Using CSSC as supporting niche cells instead of limbal fibroblasts is a much quicker process, taking only 15 days instead of 28 days, from isolation of cells to a confluent HLE layer on a minimum of 4 RAFT TEs.

Another important advantage of our method is the elimination of growth–arrested 3T3 murine fibroblasts traditionally used as feeder cells for *ex vivo* expansion of HLE^27^. This typically poses a risk as cells from a xenogenic source may carry adventitious agents into the graft. A study by Na and colleagues^28^ similarly used an *ex vivo,* xeno-feeder-free system to culture HLE. This consisted of limbal tissue explants from which an outgrowth of fibroblast-like stromal cells grew and served as an autofeeder layer for HLE cells to migrate and grow on top. In our study, cells are expanded directly on RAFT TEs so there is no requirement for pre-expansion of cells on plastic. Importantly, this approach is much more relevant to our therapeutic goal which is to transfer cells back to the patient as quickly as possible and with minimal manipulation *in vitro*. It is known that culturing cells on a tissue-like interface rather than an artificial plastic substrate helps maintain cells in their native behavioural state. Previous studies have demonstrated that cells cultured on extracellular matrix will adopt the appearance, growth characteristics and biological responses not expressed when cultured on artificial plastic or glass substrata^29^. RAFT TEs could then be used as a delivery vehicle for transplantation of HLE. In addition, clinically appropriate collagen can be used and its density can be tailored to provide optimal transparency and mechanical strength of the tissue equivalents^30^.

When manufacturing a cell-based therapy, certain safety and efficacy standards that are set by the regulating authorities must be met. This technique enables the manufacture of multiple autologous grafts, which would allow the necessary testing for safety, quality and efficacy prior to release of the graft to the patient^13^. It also helps overcome the problem of shortages in donor cornea tissue as one donor cornea can be used to isolate both epithelial and stromal cell populations.

A recent study by Basu and colleagues^31^ demonstrated successful isolation and transplantation of human limbal-biopsy derived CSSC in a mouse scar model. Further studies that investigate the use of a limbal biopsy instead of a whole corneal rim for cultivation of HLE would be greatly beneficial for the development of an autologous therapy for treatment of LESCD. In addition, investigating the ability of CSSC incorporated within RAFT TEs to support HLE growth would be beneficial for the development of an allogeneic graft, where CSSC populations could be harvested, expanded and cryo-preserved in a cell bank for future use.

A study by Sangwan and colleagues^32^ described a novel surgical technique for HLE transplantation where limbal explants were distributed evenly on amniotic membrane placed on the cornea. Their study showed short-term success with the procedure, which may have been attributed to the presence of other limbal niche cells in the explants. Our study reiterates that it may be more advantageous to replace the cells that interact in the limbal niche, including CSSC, which would support the growth of HLE.

In this study, it was found that very few stromal cells remained on the surface of RAFT TEs with some burrowing beneath the epithelial layer by the end of culture. Therefore, although the presence of CSSC is beneficial for *in vitro* expansion of HLE it may not be necessary to transplant them in large numbers for future therapeutic delivery to restore the limbal stem cell niche following ocular surface injury or disease. Future work testing the safety and efficacy of a reconstructed corneal epithelium using RAFT TEs in an animal model may confirm the clinical suitability of this method.

## Materials & Methods

### Isolation of mixed population of CSSC and HLE

Full ethical approval from the Research Ethics Committee (UK) (reference no. 10/H0106/57-11ETR10) and informed consent from all donors was obtained. All methods were carried out in accordance with the approved guidelines. Human CSSC and HLE were isolated from donor corneas as described by Du *et al.*^5^. Briefly, the superficial corneal limbal region was dissected into small fragments and digested in Dulbecco’s modified Eagle’s medium (DMEM) (Sigma-Aldrich, Dorset, UK) supplemented with 50ug/ml gentamicin (Gibco, Life Technologies, Paisley, UK), Penicillin-Streptomycin solution (1x) (Gibco, Life Technologies, Paisley, UK), containing collagenase type L (0.5 mg/ml; Sigma-Aldrich) and incubated at 37 °C and 5% CO_2_ overnight.

### Co-culture of CSSC and HLE in 2D

The resulting cell pellet containing both epithelial and stromal cells was suspended in corneal stromal stem cell (CSSC) medium consisting of a mixture of DMEM low glucose (Gibco, Life Technologies) and MCDB-201 (Sigma-Aldrich) medium, supplemented with 2% fetal bovine serum (Invitrogen, Life Technologies, Paisley, UK), 10 ng/ml epidermal growth factor (Sigma-Aldrich), 10 ng/ml platelet-derived growth factor (PDGF-BB; R&D Systems, Abingdon, Oxford, UK), Insulin-Transferrin-Selenium (ITS) solution (1x) (Gibco, Life Technologies), 0.1 mM ascorbic acid-2-phosphate (Sigma-Aldrich), 10^−8^ M dexamethasone (Sigma-Aldrich), penicillin-streptomycin solution (1x) (Gibco, Life Technologies), 50 ug/mL gentamicin (Gibco, Life Technologies), and 100 ng/mL cholera toxin (Sigma- Aldrich). This mixture was cultured in flasks coated with fibronectin-colllagen (FNC; Athena Enzyme System, Baltimore, MD, USA) at 37 °C and 5% CO_2_ until epithelial cells were confluent. CSSC media was replaced three times a week.

### Preparation of RAFT TEs

Acellular RAFT TEs were prepared in 24-well plates using rat-tail type I collagen and neutralizing solution (TAP Biosystems). Briefly, collagen solution (80% of total volume) was mixed with 10xMEM (10%), neutralizing solution (5.9%) and DMEM (4.1%). The mixture was held on ice for 30 minutes prior to seeding 2.4 ml mixture into each well. This was allowed to set for 30 minutes using an electronic heater (TAP Biosystems, Royston, Cambridge, UK) prior to gentle wicking of water from collagen hydrogels using hydrophilic porous absorbers (TAP Biosystems).

### Co-culture of CSSC and HLE in 3D

The cell suspension containing a mixed population of CSSC and HLE was divided equally onto the surface of 4 RAFT TEs (24-well) and cultured at 37 °C and 5% CO_2_ up to 13 days with CSSC media replaced daily or at least every second day.

### Fluorescein diacetate (FdA) staining of RAFT TEs: measurement of HLE growth

RAFT TEs were stained with fluorescein diacetate (FdA) at days 5, 10 and 13 during culture to assess the extent of epithelial cell growth on the surface. TEs were washed with PBS prior to incubation with FdA (10 μM final concentration) for 2 minutes in the dark at 37 °C and 5% CO_2_. FdA solution was then removed and TEs were washed with PBS twice. Photographs of the surface of TEs were taken with a camera using a yellow lens under a blue light in order to visualize the fluorescein. Following imaging, TEs were washed with phosphate buffered saline (PBS) then immersed in CSSC media and returned to culture conditions. Area of HLE growth was calculated by analyzing photographs using Image J software.

### Immunohistochemistry of RAFT TEs

RAFT TEs were assessed for phenotypic characterization of corneal stromal stem cell and epithelial cell markers following 13 days of culture. RAFT TEs were fixed with 4% paraformaldehyde for 30 minutes followed by three sequential washes in PBS. Samples for staining of CK3 (Millipore), CK8 (Chemicon International, Temecula, MA, USA) and CK15 (Santa Cruz Biotechnology) were blocked in 5% goat serum prepared in PBS with 0.5% Triton-X-100 (Sigma-Aldrich, Dorset, UK) for 60–90 minutes followed by a further wash in PBS. Goat serum (5%) prepared in 0.25% Triton-X-100 was used to block samples for p63α (Cell Signalling Technology, Danvers, MA, USA), CD73 (Abcam, Cambridge, UK) and CD90 (Abcam). 5% goat serum without Triton-X-100 was used to block samples for ABCB5 (Abcam) staining. Samples were then incubated overnight at 4 °C with the primary antibodies p63α, ABCB5, CK3, CK8, CK15 (all 1:100 dilution), CD73 or CD90 (10 μg/ml concentration). The next day, the samples were incubated with secondary goat anti-rabbit or anti-mouse 594 Alexa Fluor antibody (1:500 dilution; Invitrogen Ltd, Paisley, UK) and FITC-labelled phalloidin (1:1000 concentration; Sigma-Aldrich, Dorset, UK) for 1 hour at room temperature in the dark. Finally, samples were mounted underneath coverslips in Vectashield mounting medium containing DAPI (Vector Laboratories Inc., Burlingame, CA) and viewed and analysed on a confocal Zeiss LSM 710 microscope.

### Transmission Electron Microscopy of RAFT TEs

Following 13 days of culture, RAFT TEs with a mixed population of CSSCs and HLE were fixed in 2.5% glutaraldehyde and 2% paraformaldehyde in 0.08 M sodium cacodylate buffered to pH 7.4. Samples were then post-fixed with 1% aqueous osmium tetroxide (Elektron Technology Ltd. Essex, UK) for 2 hours at 4 °C and rinsed in distilled water. Following osmication, the samples were dehydrated through an ascending ethanol series (50%, 70%, 90% and 100%), passed through propylene oxide and infiltrated with 50:50 propylene oxide: epoxy araldite resin mixture (Elektron Technology Ltd. Essex, UK) before being embedded in full resin at 60 °C overnight. 75 nm ultra-thin sections were collected on copper grids and post-stained with lead citrate, prior to examination in a JEOL 1010 transmission electron microscope and imaged with an SC1000 Orius CCD camera (Gatan, Abingdon Oxon, UK).

## Additional Information

**How to cite this article**: Kureshi, A. K. *et al.* Human corneal stromal stem cells support limbal epithelial cells cultured on RAFT tissue equivalents. *Sci. Rep.* **5**, 16186; doi: 10.1038/srep16186 (2015).
